# Cold Plasma Controls Nitrite Hazards by Modulating Microbial Communities in Pickled Radish

**DOI:** 10.3390/foods12132550

**Published:** 2023-06-29

**Authors:** Wei Wei, Shujing Yang, Fan Yang, Xinyu Hu, Yuan Wang, Wenjun Guo, Biyue Yang, Xiang Xiao, Lin Zhu

**Affiliations:** 1 School of Agricultural Engineering, Jiangsu University, Zhenjiang 212013, China; weiwei7096@ujs.edu.cn (W.W.); 2212016008@stmail.ujs.edu.cn (S.Y.); 2212016007@stmail.ujs.edu.cn (F.Y.); 2212116064@stmail.ujs.edu.cn (W.G.); 2 School of Food and Biological Engineering, Jiangsu University, Zhenjiang 212013, China; 2211816006@stmail.ujs.edu.cn (X.H.); 18937538267@163.com (Y.W.); 2222218050@stmail.ujs.edu.cn (B.Y.); xiaoxiang1@aliyun.com (X.X.)

**Keywords:** cold plasma, pickled radish, nitrite, Gram-negative bacteria, lactic acid bacteria

## Abstract

The hazard of nitrite caused by microorganisms is the main food safety problem in the pickle production. To seek a method to control the nitrite hazards of pickles by regulating microbial community without additional substances, we focused on cold plasma because Gram-negative and Gram-positive bacteria have different degrees of sensitivity to the sterilization of cold plasma. Using radish pickles as the experimental object, based on colony counting, dynamic monitoring of pH and nitrite, qPCR and high-throughput sequencing, it was found that when the raw material was treated with dielectric barrier discharge (DBD) cold plasma at 40 kV for 60 s, Gram-negative bacteria with the potential to produce nitrite were preferentially sterilized. Meanwhile, Gram-positive bacteria dominated by the lactic acid bacteria were retained to accelerate the acid production rate, initiate the self-degradation of nitrite in advance and significantly reduce the peak value and accumulation of nitrite during the fermentation process of pickled radish. This study preliminarily verified that DBD cold plasma can inhibit the nitrite generation and accelerate the self-degradation of nitrite by regulating the structure and abundance of microbial community in radish pickles, which provides an important reference for the control of nitrite hazards in the fermentation process of pickles without additives.

## 1. Introduction

Pickles are fermented vegetable food that is crunchy and easy to prepare and is popular as a side dish in Asian countries such as China, Korea and Japan [1]. Pickles are mainly made of vegetables such as radish, pepper, cabbage, etc., with spices and salt (final concentration of 2–8% *w*/*v*) and auxiliary materials under normal temperature (20–25 °C) conditions for 6–10 days of fermentation [2]. However, pickles will generate a large amount of nitrite during the fermentation process, which will combine with nitrogen compounds in the human stomach to generate carcinogenic nitrosamines, which increase the incidence of diseases such as gastric cancer and esophageal cancer [3]. Studies have shown that the nitrite content in the fermentation broth of pickles always rises first and exceeds the Chinese national standard of 20 mg/kg and then gradually degrades to the background value by itself [4,5,6]. This is mainly due to the reduction of nitrate by Gram-negative bacteria on the surface of vegetable raw materials through nitrate assimilation or dissimilation to produce and accumulate nitrite [7,8]. As pickles continue to be fermented, lactic acid bacteria (LAB) will gradually become the dominant flora and metabolize to produce a large amount of lactic acid to reduce the pH of the fermentation broth. Low pH acidic conditions (pH < 4) both inhibit the growth of nitrite-producing Gram-negative bacteria and provide conditions for the chemical reaction of nitrite, resulting in rapid nitrite degradation [6,9,10,11]. In response to the production and degradation patterns of nitrite regulated by microorganisms, researchers have designed many strategies to control the nitrite hazards, such as the suppression of nitrite-producing Gram-negative bacteria through a variety of additives and promotion of nitrite acid degradation through direct injection of LAB strains, etc. [5,8,12,13]. However, if a strategy that does not require additional substances and starts from raw materials can be designed to control the nitrite hazards in pickles, it will provide a feasible method for food safety control in pickle processing, especially in homemade pickles.

As a new type of green sterilization technology, cold plasma has shown great potential in the preservation of agricultural products such as fruits and vegetables, meat and seafood, as well as in the production of low-molecular substances and other fields [14,15,16]. Among them, dielectric barrier discharge (DBD) cold plasma has been widely studied. It contacts the surface of microorganisms through ultraviolet photons, ions, molecules and various forms of active substances generated by ionized air, causing oxidative damage to cells and eventually leading to bacterial cell death [17]. Compared with other traditional sterilization technologies, DBD clod plasma has the advantages of a small temperature rise, low energy consumption, no pollution, no residue, short action time and easy operation [18]. In addition, when applying the DBD cold plasma to sterilize various pathogenic microorganisms in food, it was found that the sensitivity of Gram-negative bacteria to DBD cold plasma was generally higher than that of Gram-positive bacteria [18,19,20], which may be related to factors such as thicker cell walls of Gram-positive bacteria and the presence of outer membranes in Gram-negative bacteria [21]. Therefore, we hypothesize whether we could use the characteristic that different microbial groups have different sensitivities to DBD cold plasma to find an ideal sterilization treatment conditions, that is, sterilize the Gram-negative bacteria that dominate the production of nitrite in vegetable raw materials and try to retain the Gram-positive LAB that dominate the self-degradation of nitrite in pickles. In this case, it not only reduces the amount of nitrite generated during the fermentation of pickles but also accelerates the formation of a low pH environment for nitrite self-degradation, thereby controlling the nitrite hazards in pickles without adding any additives.

Therefore, this study was based on the DBD cold plasma to sterilize radish raw materials and prepare radish pickles. By comparing the colony forming unit (CFU) of the cultivable bacterial communities and LAB at different treatment times, as well as monitoring the dynamic changes of pH and nitrite content in the tested radish pickles, the optimal treatment times of DBD cold plasma were preliminarily determined. Then, combined with the results of quantitative PCR and high-throughput sequencing, we analyzed the dynamic succession process of microbial community structure and abundance of pickle fermentation under the condition of this treatment time and verified the above hypothesis of controlling the nitrite hazards in pickles based on the regulation of DBD clod plasma on Gram-negative and Gram-positive bacteria. This preliminary study helps to elucidate the feasibility of DBD cold plasma technology to solve the nitrite hazard of pickles without additives and provides new ideas for the application of DBD cold plasma to the safe preparation of fermented food.

## 2. Materials and Methods

### 2.1. Preparation and Sampling of Pickles

The raw radish samples were washed with water, drained and cut into 2 cm × 2 cm × 1 cm squares on an ultraclean bench and divided into two groups: the control group (Control) without DBD cold plasma treatment and the treated group with DBD cold plasma treatment. The treated group had a set of three sub-treatments with three different processing times of DBD cold plasma, including 30 s (T-30), 60 s (T-60) and 120 s (T-120). The radish samples of the control group were evenly divided into three parts as experimental replicates. Each sample part retained a suitable number for the count of culturable bacteria and LAB colonies, while the remaining samples were mixed with 2% sterile saline at a 1:1 (*v*/*v*) material ratio and fermented at room temperature (23 ± 2 °C). The radish samples in the treated group were evenly divided into 12 parts, and three parts were obtained for each treatment (T-30, T-60 and T-120) as experimental replicates. After DBD treatment, a suitable number of each replicate sample was retained for the same counting experiment and fermentation as the control group mentioned above. The fermentation broth of each replicate sample was taken at certain time intervals to determine the nitrite content and pH value of the samples and to extract DNA for quantitative PCR and high-throughput sequencing studies.

### 2.2. DBD Plasma Treatment

**Figure 1:**
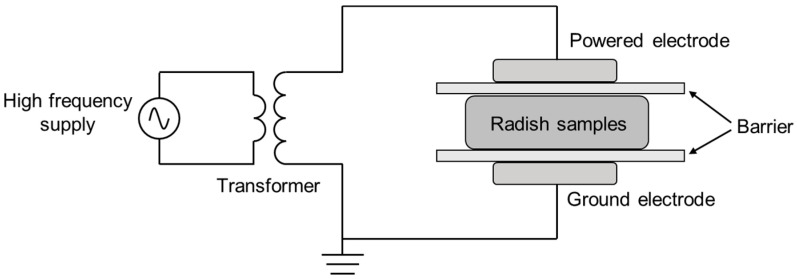
Schematic diagram of the experimental setup for the dielectric barrier discharge of cold plasma.

Plasma is generated using air as the gas source. The cold plasma sterilization equipment used in this study is a dielectric barrier discharge device. Briefly, it consists of an upper and lower aluminum electrode with a diameter of 50 mm, a glass dielectric barrier (Figure 1), a transformer and a sinusoidal power supply (CTP-2000K, Nanjing Suman Electronics Co., Ltd., Nanjing, China). The samples were placed in quartz petri dishes (diameter: 90 mm; thickness: 0.5~3 mm), which served as both a dielectric barrier (adjustable distance: 0–20 mm) and a sample reactor. Based on the DBD cold plasma device, the cut radish samples were evenly placed in a sterile glass petri dish, covered with a lid, and placed in the center of a quartz medium glass reactor. In all experiments, the gap between the interelectrode distance was maintained at approximately 15 mm, and the samples were treated for 30 s, 60 s and 120 s under the condition of air as the gas medium, a voltage intensity of 40 kV and a frequency of 20 kHz. Although the waveform of the applied voltage is not shown here, it should be noted that the waveform is a factor that can affect the characteristics of the processed products [22].

### 2.3. Counting and Analysis of Culturable Microorganisms in Raw Radish Materials

For microbial count estimation, control and treatment samples were analyzed separately. Radish (2.5 g) was homogenized in 22.5 mL of PBS buffer. The resulting suspensions were serially diluted in 0.1 M PBS buffer, and the dilutions were spread on R2A agar plates and MRS agar plates, respectively, for culturable total bacterial community and LAB colony-forming unit (CFU) count. The R2A agar plates were incubated under aerobic conditions at 37 °C for 48 h, and the MRS agar plates were incubated at 37 °C for 48 h under anaerobic conditions.

### 2.4. Monitoring of pH and Nitrite Content in Pickled Radish

The pH of the pickle fermentation broth was monitored with a pH meter (PHS-3C, Shanghai Yidian Scientific Instrument, Shanghai, China). The nitrite content in pickled radish was determined according to the naphthalene ethylenediamine hydrochloride method in the second method of GB 5009.33-2016 [23]. The pH and nitrite content were measured daily from 1 to 8 days of fermentation (when the fermentation was mature) [24].

### 2.5. Real-Time PCR Monitoring of Pickled Radish Fermentation Broth

Genomic DNA was extracted from the control group and DBD-plasma-treated pickled radish samples of the first, third, fourth and eighth day of fermentation. The pickle fermentation broth samples were centrifuged at 8000 r/min for 10 min at 4 °C. Microbial genomic DNA was extracted using the EZNA^®^ soil DNA Kit (Omega Bio-Tek, NorCross, GA, USA) following the instructions. DNA purity and concentration were checked using a NanoDrop™ One. The genomic DNA of the strain *Enterobacter asburiae* isolated from pickles was extracted as a template, and a real-time quantitative PCR system was established using primers 515F (5′-GTGYCAGCMGCCGCGGTA-3′) and 806R (5′-GGACTACHVGGGTWTCTAAT-3′) [25] designed based on the 16S V4 region. The system was used to quantify the number of bacteria in the fermentation broth of pickled radish at different fermentation stages.

### 2.6. High-Throughput Sequencing of Pickled Radish Fermentation Broth

Genomic DNA was extracted from the first-, third-, fourth- and eighth-day-fermentation broth samples of the control group and the pickled radish treated with DBD plasma. The pickle fermentation broth samples were centrifuged at 8000 r/min for 10 min at 4 °C. Microbial genomic DNA was also extracted using the EZNA^®^ soil DNA Kit (Omega Bio-Tek, NorCross, GA, USA). The DNA purity and concentration of the DNA obtained after extraction were detected by a NanoDrop TM One. The DNA sequence corresponding to the V4 region of bacterial 16S rDNA was amplified by PCR using the total DNA extracted from the sample as a template. The primers were 515F (5′-GTGYCAGCMGCCGCGGTA-3′) and 806R (5′-GGACTACHVGGGTWTCTAAT-3′) [24]. The PCR amplification system was as follows: mix system 12.5 µL, primer F1 µL, primer R1 µL, DNA template 2 µL, ddH2O to 25 µL. The amplification program was as follows: denaturation at 95 °C for 5 min; 30 cycles of denaturation at 95 °C for 30 s, annealing at 55 °C for 30 s, extension at 72 °C for 40 s; and extension at 72 °C for 10 min. PCR products were detected by electrophoresis on a 2% agarose gel.

The bacterial 16S rDNA of the pickled radish fermentation broth sample amplified by PCR was entrusted to Novogene (Beijing, China) to perform high-throughput sequencing on the Illumina-MiSeq platform to analyze the bacterial community diversity in the pickled radish samples. Chimeric sequences were removed from the Ribosomal Database Project (RDP) database using the Mothur software. The RDP classifier Bayesian algorithm was used to analyze the representative sequences of OTUs (operational taxonomic units) with a similarity level of 97%, and then the OTU species were identified based on the valid data, and the obtained OTUs were annotated. The Chao 1 index, Shannon index and Simpson index of samples were calculated by the QIIME 2 software to describe the abundance and diversity of OTUs [26].

## 3. Results

### 3.1. Effects of DBD Cold Plasma Treatment Time on the Microbial CFU Count of Raw Radish

**Figure 2:**
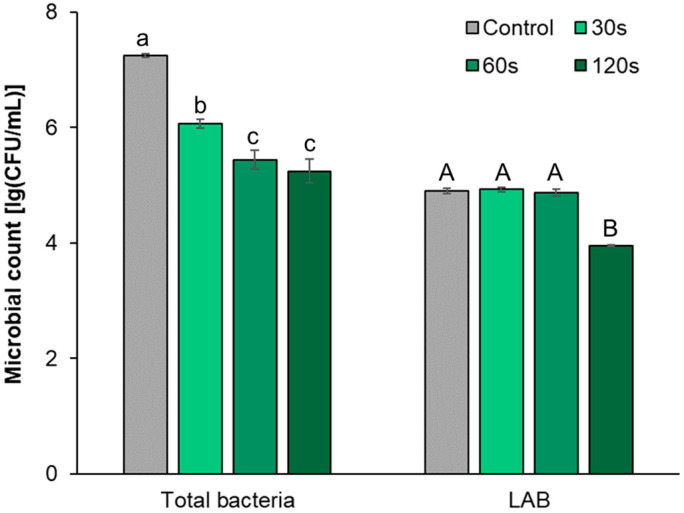
CFU count of culturable microorganisms of raw radish under different DBD cold plasma treatment times. Note: Different lowercase letters and uppercase letters represent that the differences in CFU count of culturable bacterial communities and LAB groups under different treatments, respectively, reached a significant level (*p* < 0.05).

Figure 2 shows that different treatment times of DBD cold plasma had different effects on the CFU count of bacterial community and LAB group. When the treatment time was 30 s, the total bacterial CFU count was significantly lower than that of the control group (*p* < 0.05), while the LAB CFU count did not change significantly (*p* ≥ 0.05). When the time increased to 60 s, the total bacterial CFU count continued to decrease, which was significantly lower than that of the treatment at 30 s and the control group (*p* < 0.05), while the CFU count of LAB did not change significantly (*p* ≥ 0.05). When the treatment time increased to 120 s, the total bacterial CFU count did not change significantly compared with the 60 s treatment (*p* ≥ 0.05), but the LAB CFU count began to decrease, and the number was significantly lower than that of the 60 s treatment (*p* < 0.05). The above results show that a short time (30 s~60 s) of DBD cold plasma treatment can have a greater impact on the growth of the bacterial community but less on that of the LAB group. Based on the difference in the sensitivity of Gram-negative and Gram-positive bacteria to DBD cold plasma, it is speculated that the bactericidal substances produced by cold plasma may first act on the cell wall of microorganisms, inhibiting the growth of some Gram-negative bacteria with thinner cell walls that are more sensitive to cold plasma. With the continuous increase in the treatment time (120 s), the reactive oxygen species and reactive nitrogen species generated by the plasma continuously accumulated in the cells, which aggravated the oxidative damage of the intracellular substances, resulting in serious damage to the Gram-positive LAB cells [27]. Therefore, based on the abovementioned changes of the CFU count in the bacterial community and LAB, it can be seen that under the conditions of a cold plasma working voltage of 40 kV and a treatment time of 60 s, the LAB inhabiting the radish of the pickle raw material can be well preserved, while some Gram-negative bacteria in the bacterial community may be effectively sterilized.

### 3.2. Effect of DBD Cold Plasma Treatment Time on the Dynamic Changes of pH in Pickled Radish

**Figure 3:**
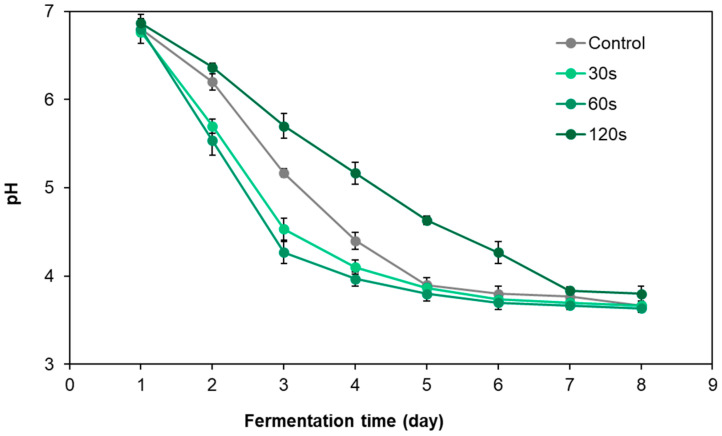
Dynamic pH changes in pickled radish with different DBD cold plasma treatment times.

The pH changes of pickled radish under different cold plasma treatment times are shown in Figure 3. During the fermentation process, the pH value of all tested pickle samples gradually decreased, but the speed and extent of the decrease showed obvious differences due to different treatment time conditions. On the second, third and fourth days of fermentation, the pH values of pickled radish samples treated with DBD cold plasma for 30 s and 60 s were significantly lower than those in the control group (*p* < 0.05). In particular, the pH value of pickled radish samples treated for 60 s (T60) was the lowest at each monitoring time point and was lower than 4 of the nitrite chemical degradation threshold on the fourth day of fermentation [28]. In contrast, the pH of the pickled radish samples treated for 120 s was higher than that of the control samples during the entire fermentation process, and the pH reached the nitrite chemical degradation threshold of 4 only on the seventh day of fermentation. The above results show that a short cold plasma treatment, especially treatment for 60 s, can significantly accelerate the pH drop of pickled radish and start the chemical acid degradation process that can control the nitrite damage of pickles one day in advance.

### 3.3. Effect of DBD Cold Plasma Treatment Times on the Self-Degradation Process of Nitrite in Pickled Radish

**Figure 4:**
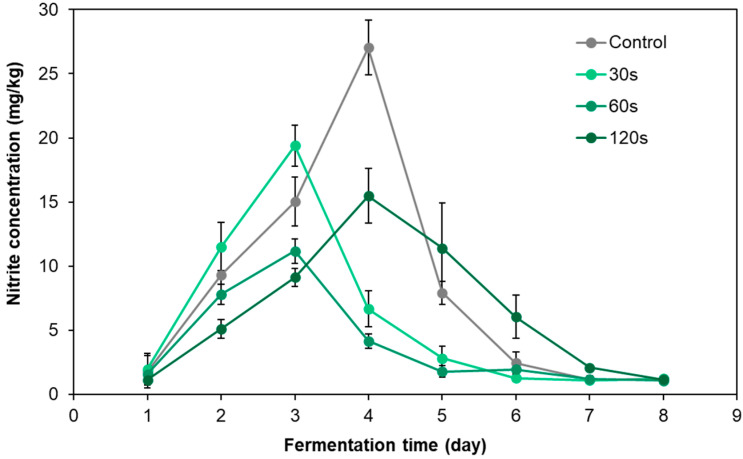
Dynamic changes in nitrite content in pickled radish under different DBD cold plasma treatment times.

Figure 4 shows the variation trend of nitrite content in pickled radish during fermentation under different cold plasma treatment times. During the fermentation process, nitrite accumulated in the pickled radish samples of the control group and peaked at 27.03 mg/kg on the fourth day of fermentation. This value exceeded the maximum limit of 20 mg/kg nitrite in pickled vegetables in China (GB 2762-2017) [29]. The peaks of nitrite in pickled radish samples treated by DBD clod plasma for 30 s and 60 s both appeared prior to the third day, and the peak values were 19.4 mg/kg and 11.2 mg/kg, which were lower than the Chinese safety standards, while the total accumulation of nitrite during fermentation decreased to 66.9% and 42.6% of the control group, respectively. In addition, the peak of nitrite in pickled radish samples treated for 120 s still appeared on the fourth day of fermentation. Although the peak nitrite was 13.2 mg/kg, which was lower than the Chinese safety standard, the total accumulation of nitrite only dropped to 76.1% of the control group. The above results show that all of different DBD cold plasma treatment times can reduce the peak value and total accumulation of nitrite in pickled radish. Among them, the pickled radish samples treated with DBD cold plasma for 60 s showed the best results in terms of the time to reach the nitrite peak, the extent to which the nitrite peak can be reached and the total amount of accumulated nitrite.

In summary, we believe that under the conditions of a DBD cold plasma operating voltage of 40 kV and a treatment time of 60 s, the LAB group inhabiting the raw material of pickled radish may be well preserved, leading to a significant acceleration of pH decline in pickled radish. Such behavior of the LAB group initiated the chemical acid degradation process of nitrite that can control the nitrite hazards in pickled radish in advance, and achieved the optimal control effect of nitrite hazards in all tested DBD cold plasma treatments.

### 3.4. Effects of DBD Cold Plasma on the Microbial Community Abundance in Pickled Radish

**Figure 5:**
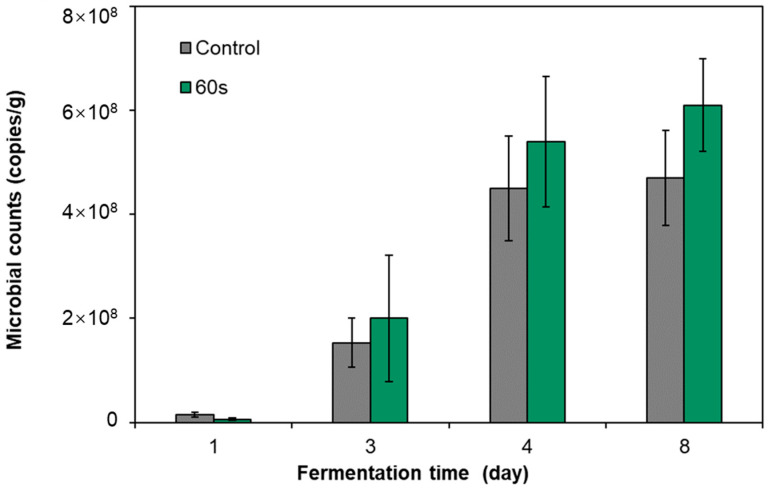
Effects of DBD cold plasma treatment for 60 s on bacterial 16S rRNA abundance in pickled radish fermentation broth.

The previous study confirmed the feasibility of using DBD cold plasma to treat pickled radish raw materials to control nitrite hazards. In order to further validate our proposed idea of using DBD to sterilize Gram-negative bacteria that produce nitrite and retain the LAB that dominate nitrite self-degradation, we analyzed the microbial community characteristics of pickled radish treated by DBD cold plasma. Therefore, quantitative PCR technology was first used to monitor the bacterial community abundance of pickled radish in the treated group (treated for 60 s) and the control group (Figure 5). On the first day of fermentation, the abundance of bacterial 16S rRNA sequences in the treated group was lower than that in the control group but had no significant difference (*p* ≥ 0.05). From the third day to the eighth day of fermentation, the abundance of bacterial 16S rRNA sequences in the treated group was higher than that in the control group but had no significant difference (*p* ≥ 0.05). The results showed that after the raw materials of pickles were sterilized by DBD cold plasma, the ecological niche left by the sterilized bacteria would be quickly filled by the remaining bacteria, even to a higher than the original level.

### 3.5. Effects of DBD Cold Plasma on the Microbial Community Composition of Pickled Radish

To further identify the bacterial community composition sterilized and retained by DBD cold plasma for different treatment times, high-throughput sequencing of bacterial 16S rRNA gene sequence fragments was carried out using the bacterial universal primers used in the above qPCR experiments. Based on the obtained high-throughput sequencing library data with high coverage, OTUs for each treatment and associated species richness and diversity indices were determined (Table 1). At the four time points monitored, the OTU and Chao1 index of the treated group (treated for 60 s) were always lower than those of the control group, and the reduction degree of the Shannon index was also lower than that of the control group. However, the treated group maintained a consistently higher Simpson index. The results showed that after DBD cold plasma treatment, the number of bacterial species inhabiting pickled radish was greatly reduced and showed a lower level of structural diversity, but the dominance of dominant species was obvious.

**Figure 6:**
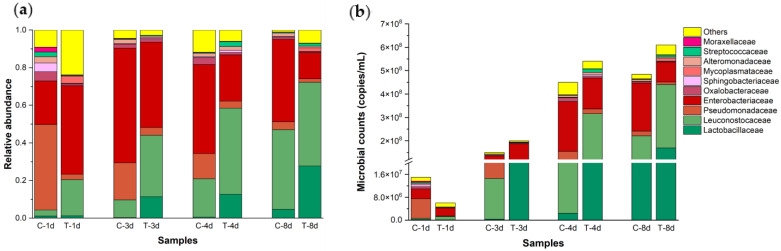
Effects of DBD cold plasma treatment for 60 s on the relative abundance (**a**) and absolute abundance (**b**) of the bacterial community composition of pickled radish at the family level. Note: C-1d, C-3d, C-4d and C-8d are pickled radish samples collected from the control group on the 1st, 3rd, 4th and 8th days of fermentation; T-1d, T-3d, T-4d and T-8d are pickled radish samples collected from samples treated by DBD low-temperature plasma for 60 s on the 1st, 3rd, 4th and 8th days of fermentation.

Continued species annotation for high-throughput sequenced OTUs. Species annotation was performed at the phylum, class, order, family and genus taxonomic levels of bacteria, and the corresponding annotation effects were evaluated. The results showed that the proportion of OTUs with unannotated results at the genus level was relatively large (38–64%), which was not suitable for comparative analysis between treatments (data not specifically listed). However, each treatment had a higher OTU annotation rate at the family level, and only 3–11% of the OTUs were not annotated, which is more suitable for further comparative analysis. The Top 10 groups at the family level for each treatment included *Enterobacteriaceae*, *Pseudomonadaceae*, *Oxalobacteraceae*, *Alteromonadaceae*, *Moraxellaceae*, *Sphingobacteriaceae* and *Mycoplasmataceae* for Gram-negative bacteria and *Leuconostocaceae*, *Lactobacillaceae* and *Streptococcaceae* for Gram-positive bacteria (Figure 6a). On the first day of fermentation, the relative abundance of Gram-negative bacteria decreased from 84.1% to 55.4% after cold plasma treatment, and the LAB flora increased from 6.9% to 20.7%. On the third day of fermentation, the relative abundance of Gram-negative bacteria decreased from 85.6% to 52.8%, and the LAB group increased from 10.0% to 44.5%. On the fourth day of fermentation, the relative abundance of Gram-negative bacteria decreased from 66.8% to 32.9%, and the LAB group increased from 21.3% to 61.1%. On the eighth day of fermentation, the relative abundance of Gram-negative bacteria decreased from 51.4% to 19.4%, and the LAB group increased from 47.6% to 73.7%. The above results clearly demonstrate that DBD cold plasma can change the microbial community composition of pickled radish and promote Gram-positive LAB to replace Gram-negative bacteria to dominate the pickle microbial community.

We combined the results of the relative abundance of bacterial communities at the family level with the quantitative PCR results above and displayed the bacterial community structure composition in the form of absolute abundance (Figure 6b). On days 1, 3, 4 and 8 of fermentation, the total abundance of Gram-negative bacteria was 1.3 × 10^7^, 1.3 × 10^8^, 3.0 × 10^8^ and 2.4 × 10^8^ (copies/mL) in the control, respectively, while in the samples treated for 60 s, the abundance of Gram-negative bacteria was 3.3 × 10^6^, 1.1 × 10^8^, 1.8 × 10^8^ and 1.2 × 10^8^ (copies/mL), respectively, which was lower than the values of the control group in the same fermentation period. The total abundance of Gram-positive LAB was 1.2 × 10^6^, 1.5 × 10^7^, 9.6 × 10^7^ and 2.2 × 10^8^ (copies/mL) in the control treatment, while in the samples treated for 60 s, they were 1.0×10^6^, 8.9 × 10^7^, 3.3 × 10^8^ and 4.5 × 10^8^ copies/mL, respectively. It is worth noting that in the samples treated for 60 s, the *Leuconostocaceae* belonging to LAB group increased to 235% of the control group on the first day of fermentation, while the *Lactobacillaceae* and *Streptococcaceae*, similar to other Gram-negative bacteria, showed a decreasing state. These results indicate that when using DBD cold plasma to treat pickled radish raw materials, Gram-negative bacteria can indeed be largely sterilized, while Gram-positive LAB can be retained, especially the *Leuconostaceae* family. These LAB replaced the initially dominant Gram-negative bacteria since the fermentation of radish pickles, leading to a rapid decline in pickle pH and effective control of nitrite hazards.

## 4. Discussion

Based on the difference in sensitivity between Gram-negative and Gram-positive bacteria to DBD cold plasma, this paper applies DBD cold plasma to the study of nitrite reduction in radish pickles. The aim of this study is to eliminate the Gram-negative bacteria that dominate nitrite production and maximize the retention of Gram-positive LAB that can degrade nitrite and thus form a nitrite hazard control for radish pickles. Under a fixed voltage, the samples treated with DBD cold plasma for 60 s showed a higher reduction rate of nitrite peak and accumulation. Under this condition, the abundance of Gram-negative bacteria, mainly *Enterobacteriaceae*, *Pseudomonas*, *Oxalobacteraceae*, *Altermonas* and *Sphingobacteriaceae*, in the bacterial community inhabited by raw radish significantly decreased. These bacterial families include bacterial genera that have been shown to inhabit pickles and have nitrite-producing potential, such as *Enterobacter* and *Klebsiella* of *Enterobacteriaceae*, *Pseudomonas* of *Pseudomonadaceae*, *Alteromonas* of *Alteromonadaceae* and *Sphingobacterium* of *Sphingobacteriaceae* [30,31,32,33,34]. At the same time, a large amount of LAB of *Leuconostocaceae* belonging to Gram-positive bacteria was retained. *Leuconostocaceae* replaced *Pseudomonadaceae* as the second most dominant flora at the beginning of fermentation, and then its abundance continued to increase, replacing *Enterobacteriaceae* as the most dominant flora. The metabolic acid production from *Leuconostocaceae* may lead to a rapid decrease in the pH of the fermentation broth, forming an acidic environment to control the nitrite hazard of radish pickles. Therefore, DBD cold plasma can achieve an ideal control effect of nitrite damage in pickles by changing the microbial community structure. However, for fermented vegetables, the microbial community composition was closely related to pickle quality [6,35,36]. Therefore, changes in microbial composition in the fermentation system may lead to changes in the basic physical and chemical indicators, nutrition or flavor of mature kimchi. Previous research reports have also pointed out that improper low-temperature plasma treatment may also have beneficial or harmful effects on the physical and chemical properties of food, such as nutrition, color or texture [14,37]. In addition, there is little research on whether cold plasma treatment can lead to toxic compounds. Therefore, to determine the effective dose without any harmful effects, subsequent monitoring of the quality of vegetables after cold plasma treatment and the finished pickles after fermentation is a follow-up study. This can better help us to evaluate the application potential of cold plasma in the preparation of fermented vegetables, thus making it more suitable for application in the fermented vegetable preparation industry.

In recent years, cold plasma as a green technology to sterilize various food-borne pathogens is gradually being promoted in the field of food processing. In some studies that focus on fruits, meat, seafood and other preservation objects, in order to achieve better sterilization effects, the working voltage of the cold plasma is usually controlled between 60 and 75 kV. However, Zhao et al. [38] found that when packaged fermented vegetables were treated with DBD plasma with a voltage of 60 kV and a frequency of 50 Hz for 180 s, the nitrite content of the treated vegetables was higher than that of untreated pickles. When studying the plasma-active water interaction, Liu et al. found that the short-lived RNS generated by the plasma discharge is converted to nitrite or nitrate [39]. It can be seen that the high-power cold plasma treatment of vegetable raw materials may lead to the production of nitrite [40]. Therefore, in this study, we fixed the working voltage at a low 40 kV and dried the skin moisture of the raw radish before the cold plasma treatment, preventing the possibility that the plasma-active molecules react with the moisture to generate nitrite. At the same time, along with the fermentation of pickled radish, the retained *Leuconostocaceae* of LAB in the fermentation system rapidly metabolized and produced acid to control the production and accumulation of nitrite. Therefore, even if the plasma treatment with a voltage of 40 kV produces trace amounts of nitrite, it can be completely degraded by microorganisms and acid environment in the later stage of fermentation without affecting the safety of the pickled product. Hou et al. [41] also found that the content of nitrite increased in a short period of time and then automatically decreased when fermenting radish raw materials with cold plasma and believed that cold plasma reduced the growth of yeast by inhibiting the growth of yeast. The production of nitrite, but its internal mechanism has not been deeply analyzed. However, based on the results of high-throughput sequencing in this study, we believe that compared with pickle yeast, *Enterobacter*, *Klebsiella*, *Pseudomonas* and *Altermonas* at the initial stage of fermentation and other gram-negative bacteria that can reduce nitrate to produce nitrite may play a more decisive role in the production and accumulation of nitrite in pickles. However, a more accurate understanding of the microbiological mechanisms underlying the regulation of nitrite growth and decline during the fermentation process of pickles by DBD cold plasma still needs to be explored in depth, in order to provide a theoretical basis for the application of DBD cold plasma to solve the problem of nitrite harm in fermented vegetables.

## 5. Conclusions

Based on the different sensitivities of different microorganisms to DBD cold plasma sterilization, this study proposes the idea that DBD cold plasma can control the harm of nitrite in pickled vegetables by regulating the microbial community structure. Preliminary validation was conducted on pickled radish. Under fixed DBD cold plasma working voltage conditions (U = 40 kV), the analysis and comparison of microbial numbers, pH values and nitrite changes in radish samples treated with cold plasma for 30 s, 60 s and 120 s showed that cold plasma treatment for 60 s could sterilize 98% of the cultivable bacteria inhabiting the raw radish while retaining 94% of the cultivable LAB group. The pickled radish treated for 60 s had the fastest rate of pH reduction, enabling nitrite to initiate acid degradation one day earlier and achieving the best control effect on nitrite peak and accumulation, which decreased to 41.3% and 42.6% of the control, respectively. Based on qPCR and high-throughput sequencing, it was analyzed that after 60 s of cold plasma treatment, the abundance of Gram-negative bacteria with nitrite production potential represented by *Pseudomonasceae* and *Enterobacteriaceae* decreased by 41–74% compared with the control treatment. At the same time, the abundance of Gram-positive LAB, mainly *Leuconostocaceae*, that can quickly produce acid and initiate nitrite degradation, increased to 1.2 to 6 times that of the control group. In summary, we have preliminarily verified that through different plasma sensitivity levels of Gram-negative and positive bacteria, DBD cold plasma may prioritize the sterilization of Gram-negative bacteria that produce nitrite while retaining Gram-positive LAB that can promote acid degradation of nitrite. It is a feasible green measure to control the harm of nitrite in pickles, providing an important reference for establishing a nitrite hazard control system without any additives in pickles.

## Figures and Tables

**Table 1:** OTU-based species and bacterial community structural diversity of high-throughput sequencing libraries.

Treatments	Total Reads	OTU	Chao1	Shannon	Simpson	Coverage
C-1d	81,573	394	401.7	4.4	0.8	0.998
C-3d	89,664	405	470.1	3.6	0.8	0.998
C-4d	65,255	353	325.5	3.5	0.8	0.993
C-8d	54,884	336	338.6	3.6	0.9	0.999
T-1d	61,977	368	361.6	4.0	0.9	0.998
T-3d	88,872	378	365.3	3.8	0.9	0.998
T-4d	83,706	323	311.0	3.7	0.9	0.998
T-8d	89,907	300	293.0	3.6	0.9	0.998

Note: C-1d, C-3d, C-4d and C-8d are pickled radish samples collected from the control group on the 1st, 3rd, 4th and 8th days of fermentation; T-1d, T-3d, T-4d and T-8d are pickled radish samples collected from samples treated with DBD low-temperature plasma for 60 s on the 1st, 3rd, 4th and 8th days of fermentation.

## Data Availability

The data presented in this study are available on request from the corresponding author.
